# Functionality of promoter microsatellites of arginine vasopressin receptor 1A (AVPR1A): implications for autism

**DOI:** 10.1186/2040-2392-2-3

**Published:** 2011-03-31

**Authors:** Katherine E Tansey, Matthew J Hill, Lynne E Cochrane, Michael Gill, Richard JL Anney, Louise Gallagher

**Affiliations:** 1 Neuropsychiatric Genetics Research Group, Department of Psychiatry, Institute of Molecular Medicine, Trinity College Dublin, Dublin, Ireland

## Abstract

**Background:**

Arginine vasopressin (AVP) has been hypothesized to play a role in aetiology of autism based on a demonstrated involvement in the regulation of social behaviours. The arginine vasopressin receptor 1A gene (*AVPR1A*) is widely expressed in the brain and is considered to be a key receptor for regulation of social behaviour. Moreover, genetic variation at *AVPR1A *has been reported to be associated with autism. Evidence from non-human mammals implicates variation in the 5'-flanking region of *AVPR1A *in variable gene expression and social behaviour.

**Methods:**

We examined four tagging single nucleotide polymorphisms (SNPs) (rs3803107, rs1042615, rs3741865, rs11174815) and three microsatellites (RS3, RS1 and AVR) at the *AVPR1A *gene for association in an autism cohort from Ireland. Two 5'-flanking region polymorphisms in the human *AVPR1A*, RS3 and RS1, were also tested for their effect on relative promoter activity.

**Results:**

The short alleles of RS1 and the SNP rs11174815 show weak association with autism in the Irish population (*P *= 0.036 and *P *= 0.008, respectively). Both RS1 and RS3 showed differences in relative promoter activity by length. Shorter repeat alleles of RS1 and RS3 decreased relative promoter activity in the human neuroblastoma cell line SH-SY5Y.

**Conclusions:**

These aligning results can be interpreted as a functional route for this association, namely that shorter alleles of RS1 lead to decreased *AVPR1A *transcription, which may proffer increased susceptibility to the autism phenotype.

## Background

The neuropeptide arginine vasopressin (AVP) has been hypothesized to play a role in the aetiology of autism based on a demonstrated involvement in social bonding and in the regulation of a variety of socially relevant behaviours in animal models. Pharmacological experiments in rodents have shown a role for vasopressin in learning and memory, aggression and affiliative behaviours. Increases in AVP are associated with stressful or defensive circumstances and AVP is also involved in aggressive behaviours and pair bonding, mostly in males [1]. AVP regulates male social behaviour not just through higher expression in males but also in steroid-sensitive brain sexual dimorphisms in AVP neurons [1,2]. AVP may therefore influence sexually dimorphic social behaviours in a range of species [3]. The role of sex hormones on AVP is of interest in the context of autism considering that the ratio of affected males with autism compared to affected females is highly skewed (4:1) [4]. Only one study has examined the relationship between AVP levels and autism; Boso *et al. *[5] found AVP levels to be higher in plasma from individuals with autism than controls (*P *= 0.02).

AVP acts through binding to G-protein-coupled receptors. One of these receptors, arginine vasopressin receptor 1A (AVPR1A), is widely expressed in the brain. There is considerable evidence to implicate the *AVPR1A *gene in social behaviours. *AVPR1A*-knockout mouse models show impaired social recognition and decreased anxiety behaviours, while overexpression of *AVPR1A *in mice resulted in increased social memory [6,7]. Disruption in *AVPR1A *expression in the brain has been associated with major behavioural changes [8-11]. In non-human mammals, the variable expression of *AVPR1A *is implicated in changes in social bonding, parental rearing behaviours and social recognition and memory. Hammock and Young [12] described behavioural differences in voles (genus *Microtus*) that was related back to the AVP neuropeptide. Prairie voles (*Microtus ochrogaster*) are socially monogamous through multiple breeding seasons with both the males and females contributing to rearing of the young. These behaviours are not typically seen within closely related species such as the montane vole (*Microtus montanus*) and meadow vole (*Microtus pennsylvanicus*). This phenomenon has been attributed to variable expression resulting from a microsatellite marker in the 5'-flanking region of the *AVPR1A *gene in *Microtus *[13,14].

**Figure 1:**
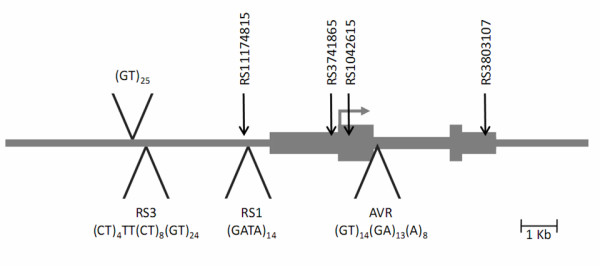
**Schematic of the arginine vasopressin receptor 1A gene (*AVPR1A*)**.

In humans, the 5' region of the gene contains four repeat elements; three promoter repeats ([GT]_25_, RS1, ([GATA]_14_) and RS3 ([CT]_4_TT[CT]_8_[GT]_24_)) and an intronic repeat (AVR ([GT]_14_[GA]_13_[A]_8_)) (see Figure 1) [15]. These polymorphisms, specifically RS1 and RS3 have been studied extensively in terms of their relationship to autism. The data reported thus far has produced inconsistent association with different alleles and markers implicated at this gene. In a sample of 115 autism spectrum disorder (ASD) proband-parent trios, Kim *et al. *[16] did not observe association for alleles of RS1 or RS3. In a smaller sample of 65 ASD trios, Wassink *et al. *[17] reported modest association with a shorter allele of the RS1 polymorphism being undertransmitted to probands with autism (max *P *= 0.008). This was not confirmed in a study of 128 individuals with ASD by Yirmiya *et al. *[18], where association was observed at the AVR polymorphism (max *P *= 0.003). More recently, Yang *et al. *[19] identified association at both RS1 and RS3 in a Korean ASD family-based study. However, the association at the RS1 markers was not consistent with previous findings, which may reflect population differences.

Various studies have linked different lengths of the *AVPR1A *polymorphisms to behavioural traits in non-clinical samples. Knafo *et al. *[20] reported that subjects with shorter length RS3 polymorphisms were found to be less generous by the 'dictator game' indicating less altruistic behaviour [20]. Moreover, shorter variants in general were associated with lower levels of *AVPR1A *mRNA in postmortem hippocampus. Recent neuroimaging approaches have also indicated a link between gene expression levels and amygdala activation [21].

Recent studies have highlighted the role of rare *de novo *inherited copy number variants in the aetiology of autism [22]. However, only rare *de novo *and inherited variants are established as genetic risk factors, and these account for a small proportion of the total genetic risk [23]. Furthermore, in a recent paper from the International Schizophrenia Consortium, common genetic variants accounted for over 30% of the genetic risk for schizophrenia [24] and there is no reason to believe this will not be the case for autism as well. The genetic factors influencing the aetiology of autism are likely to be a combination of rare, private and more common variants. The action of which are not likely to be mutually exclusive.

Based on prior evidence suggesting a role for AVP in human and animal social cognition, and prior reports of genetic association between variants in vasopressin-related genes and autism, we hypothesized that variants in *AVPR1A *would be associated with autism in our Irish autism sample. We examined the variants RS1, RS3, AVR and four tagging single nucleotide polymorphisms (SNPs) across the gene locus, namely rs3803107, rs1042615, rs3741865 and rs11174815, for association with autism in the Irish autism trio collection. Furthermore, considering the evidence to support a role of 5'-flanking variation in the expression of *AVPR1A *in *Microtus*, we hypothesised that variation in the 5'-flanking region of the human *AVPR1A *gene at the microsatellites' RS3 and RS1 polymorphisms would influence the relative effects on promoter activity.

## Methods

### Sample description

A total of 177 families, consisting of an affected child and both parents were recruited through schools, parent support groups and clinician referral. The male to female ratio was 4.68:1. Autism diagnoses were confirmed using Autism Diagnostic Interview - Revised (ADI-R) [25] and the Autism Diagnostic Observation Schedule - Generic (ADOS-G) [26]. IQ and Vineland Adaptive Behaviour Score (VABS) [27] were evaluated to establish a distribution of functioning within the sample. Individuals with known medical causes of autism (for example, tuberous sclerosis, extreme prematurity, congenital rubella) were excluded. Subjects met the ADI-R criteria for autism, and the ADOS-G criteria for autism or ASD. All individuals had an IQ >35 or a mental age (as defined by the VABS) >18 months. Ethical approval was obtained from the Health Services Executive Linn Dara Child and Adolescent Psychiatry Ethics Committee, Ireland. DNA was extracted from blood or buccal swabs using the phenol/chloroform extraction method.

### Genotyping

Using the HapMap CEPH data, four SNPs in the *AVPR1A *locus were identified using the tagger function of Haploview [28]. SNPs were selected using pairwise tagging with an r^2 ^threshold of 0.8 with an additional 2000 base pairs taken 5' and 3' of the gene transcript. SNP genotyping was performed by a commercial genotyping company, KBiosciences (http://www.kbioscience.co.uk/) using Kaspar assays. Three additional microsatellite polymorphisms (RS1, RS3, and AVR) at the *AVPR1A *gene locus were also genotyped. RS1: forward (fluorescent) 5'-AGG GAC TGG TTC TAC AAT CTG C-3'; reverse 5'-ACC TCT CAA GTT ATG TTG GTG G-3'. RS3: forward (fluorescent) 5'-CCT GTA GAG ATG TAA GTC CT-3'; reverse 5'-TCT GGA AGA GACT TAG ATG G-3'. AVR: forward (fluorescent) 5'-ATC CCA TGT CCG TCT GGA C-3'; reverse 5'-AGT GTT CCT CCA AGG TGC G-3' [15,18]. Each reaction contained 0.5 mM primer. The sample was initially heated for 10 min at 95°C followed by 30 cycles of 95°C (30 s), 55°C (30 s), 72°C (40 s) and a final extension step of 72°C for 10 min. An ABI 3130XL Genetic Analyzer (Applied Biosystems, Foster City, CA, USA) was used to determine the length of each PCR product. Samples were analysed using Genemapper v2.7 (Applied Biosystems).

### Construction of the reporter vectors

**Figure 2:**
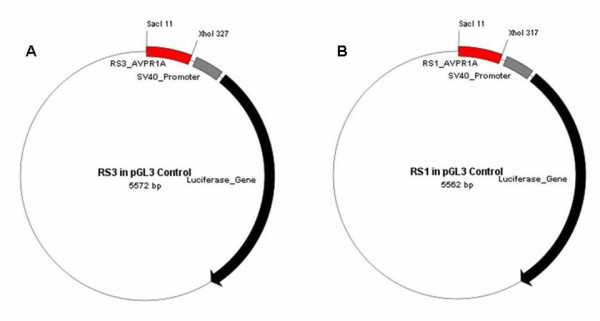
**Vector maps of the RS3 (a) and RS1 (b) alleles subcloned into pGL3 control vector**. Inserts were chosen based upon length of polymorphism, two per polymorphism. The restriction sites used for both RS3 and RS1 were *Sac*I and *Xho*I, located before the viral promoter SV40.

DNA samples of individuals homozygous for the RS3 variants (allele 325 and allele 339) and RS1 (allele 306 and 322) were selected from CEPH HapMap individuals. PCR amplification of a DNA fragment containing allele 325 and allele 339 of RS3 were amplified using primers: forward 5'-CCT GTA GAG ATG TAA GTG CT-3'; reverse 5' CTC GAG TCT GGA AGA GAC TTA GAT GG-3'. For RS1, allele 306 and allele 322 were amplified using primers: forward 5'-AGG GAC TGG TTC TAC AAT CTG C-3'; reverse 5'-CTC GAG ACC TCT CAA GTT ATG TTG GTG G-3'. Amplified sequences were cloned into the pGEM-T vector (Promega, Southampton, UK). The RS3 and RS1 alleles were subcloned into the *Sac*I and *Xho*I site in the pGL3 control vector (Promega). The amplified promoter fragments do not contain the core promoter elements of *AVPR1A*. Therefore, the pGL3 control vector as opposed to the pGL3 Basic vector was used to examine the modulating influence of these elements as 5'-flanking polymorphism through an SV40 promoter. Vector maps of the tested constructs are shown in Figure 2. Sequence analysis using a BigDye Terminator v3.1 Cycle Sequencing Kit (ABI PRISM, Applied Biosystems) was performed to confirm the fidelity and orientation of all inserts.

### Cell culture and transfection

The human neuroblastoma cell line SH-SY5Y, were maintained in RPMI 1640 containing 10% foetal bovine serum (FBS) at 37°C and 5% CO_2_. Transfections were performed in triplicate a minimum of three times using Lipofectamine 2000 reagent (Invitrogen, Carlsbad, CA, USA) according to the manufacturer's instructions.

### Luciferase assay

Luciferase assays were performed using the dual-glow luciferase system (Promega) according to the manufactures' instructions. Luminescence was measured on a Victor plate reader (PerkinElmer, Boston, MA, USA). Transfection efficiency was corrected for by normalising data relative to the cotransfected renilla luciferase pRL-cytomegalovirus (CMV) control vector (Promega). For the RS3 and RS1 constructs, 100 ng of test constructs were cotransfected with 500 ng pGL3 control vector. Expression was reported as relative to the pGL3 control vector.

### Statistical analyses

Transmission disequilibrium test (TDT) for the three microsatellites from *AVPR1A *were analysed in UNPHASED [29]. Each allele is tested independently for transmission disequilibrium. Research previously undertaken in voles demonstrated the repeat length of the polymorphism modified gene expression, transcriptional pattern in the brain and social behaviours [14]. Therefore, we decided to test for association between the length of polymorphic repeat and autism. Alleles were grouped in 'long' alleles and 'short' alleles for RS1, RS3 and AVR polymorphisms. Nomenclature of alleles for RS1 and RS3 can be found in Yirmiya *et al. *[18]. For RS3, 'long' refers to allele 327 and all repeats longer than 327 and 'short' refers to allele 325 and all repeats shorter than allele 325 consistent with the previously published study of Knafo *et al. *[20]. For RS1, 'long' consists of allele 314 and all repeats larger than allele 314 and 'short' consists of allele 310 and all repeats shorter than allele 310. For AVR, 'short' includes allele 213 and all repeats shorter than allele 213, while 'long' includes allele 215 and all repeats larger than allele 215. A similar procedure has been applied to other complex polymorphism, such as the nitric oxide synthase 1 (NOS1) ex1f- variable number tandem repeat (VNTR) [30-33].

Hardy-Weinberg equilibrium and TDT analyses for SNP markers were performed using PLINK [34]. TDT for dichotomised microsatellites (long versus short) was also performed using PLINK. No Mendelian incompatibilities were detected and all variants were found to be in Hardy-Weinberg equilibrium (*P *> 0.01) in the parents. Permutation procedures were performed in PLINK using the mperm function (*n *= 1000). Haplotypes were defined using Gabriel's confidence intervals and tested for association using permutation in Haploview (*n *= 1000) [28,35].

The statistical significance of differences in relative gene reporter activity was examined using two-tailed Student's *t *test in Stata v. 10 (StataCorp, College Station, TX, USA).

### Power calculation

In the Irish autism sample (*n *= 177) at minor allele frequencies of 0.20, there was greater than 80% power to observe an effect of odds ratio (OR) approximately 1.35 at an uncorrected *P *= 0.05 level. This calculation considers a modest effect size and also no linkage disequilibrium between the test and causative marker.

## Results

### Genetic association analyses

Of the seven markers (four SNPs and three microsatellite markers), only one showed a significant association with autism in the Irish autism sample (rs11174815, nominal *P *= 0.008). Dichotomisation of the RS1 allele revealed a weak association of the RS1 short allele and autism (nominal *P *= 0.036; odds ratio (OR) 1.44; 95% confidence interval 1.02 to 2.04) (Tables 1 and 2). No significant haplotype associations were observed (data not shown).

**Table 1:** Genotype association results using UNPHASED for multiallelic markers and PLINK for dichotomous markers

Marker	Allele	Frequency	T	NT	χ^2^	*P *value
RS3	305	0.001	0	1	1.00	0.32
	
	311	0.005	2	1	0.33	0.56
	
	319	0.001	0	1	1.00	0.32
	
	321	0.008	3	1	1.00	0.32
	
	323	0.058	19	16	0.26	0.61
	
	325	0.102	31	24	0.89	0.35
	
	327	0.214	45	36	1.00	0.32
	
	329	0.210	43	54	1.25	0.26
	
	331	0.099	25	26	0.02	0.89
	
	333	0.167	44	42	0.05	0.83
	
	335	0.026	6	11	1.47	0.23
	
	337	0.014	4	2	0.67	0.41
	
	339	0.059	14	20	1.06	0.30
	
	341	0.015	4	5	0.11	0.74
	
	343	0.017	5	6	0.09	0.76
	
	345	0.004	2	1	0.33	0.56
	
	Short	0.390	86	65	2.92	0.09

RS11174815	**A**	**0.015**	**0**	**7**	**7.00**	**0.008^a ^0**

RS1	302	0.004	0	3	3.00	0.08
	
	306	0.161	39	31	0.91	0.34
	
	310	0.448	67	52	1.89	0.17
	
	314	0.193	33	46	2.14	0.14
	
	318	0.079	20	26	0.78	0.38
	
	322	0.096	24	30	0.67	0.41
	
	330	0.014	5	1	2.67	0.10
	
	334	0.005	2	1	0.33	0.56
	
	**Short**	**0.518**	**78**	**54**	**4.36**	**0.036^b^**

RS3741865	T	0.008	2	1	0.33	0.56

RS1042615	T	0.436	55	58	0.08	0.78

AVR	204	0.002	1	0	1.00	0.32
	
	208	0.010	2	0	2.00	0.16
	
	210	0.108	16	21	0.68	0.41
	
	212	0.272	43	41	0.05	0.83
	
	214	0.461	51	48	0.09	0.76
	
	216	0.040	10	10	0.00	1.00
	
	218	0.006	0	1	1.00	0.32
	
	220	0.064	10	11	0.05	0.83
	
	222	0.001	0	1	1.00	0.32
	
	Short	0.411	79	79	0.00	1.00

RS3803107	T	0.178	32	30	0.06	0.79

T = transmitted, NT = non-transmitted.

Associations at the suggestive α level 0.05 are shown in bold text.

^a^Empirical *P *= 0.01. ^b^Empirical *P *= 0.08.

**Table 2:** Multimarker transmission disequilibrium test (TDT) for the microsatellite markers RS3, RS1 and AVR reporting the likelihood ratio statistic (LRS), degrees of freedom (df) and global *P *value from UNPHASED

Marker	LRS	df	*P *value
RS3	10.76	15	0.769

RS1	12.22	7	0.094

AVR	8.085	8	0.425

### Gene reporter assays for the RS1 and RS3 polymorphism of AVPR1A

**Figure 3:**
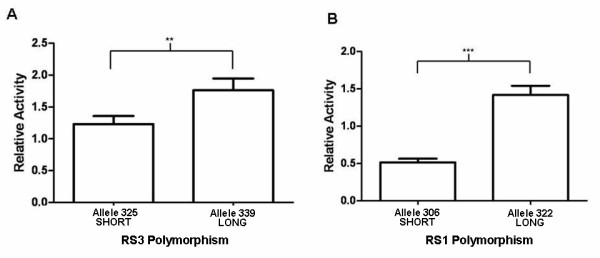
**Relative activity of the RS3 (a) and RS1 (b) polymorphisms constructs in the neuronal SH-SY5Y cell line**. (a) Longer length repeat of the RS3 polymorphism of the arginine vasopressin receptor 1A gene (*AVPR1A*) showed higher relative activity compared to the shorter length repeat of RS3 in SH-SY5Y cell lines (*P *= 0.0081, Student's *t *test). (b) Longer length repeat of the RS1 polymorphism of *AVPR1A *showed higher relative activity compared to the shorter length repeat (*P *= 0.0005, Student's *t *test). **P < 0.01; ***P < 0.001. The graph shows the mean of the data and standard error of the mean.

Differential activity was observed for alleles of both the RS1 and RS3 variants when expressed as a luciferase construct in the pGL3 control vector. In SH-SY5Y cells, the long repeat of the RS1 polymorphism showed 2.7 times higher relative activity compared to the shorter length repeat (*P *= 0.0005, Student's *t *test) (Figure 3). Similarly, for the long repeat of the RS3 variant was 1.4 times more active than the short-repeat allele (*P *= 0.0081, Student's *t *test) (Figure 3).

## Discussion

In the current work we present the results of genetic investigations of the *AVPR1A *gene in an Irish autism sample. We followed association analysis with investigations to examine the role of promoter region polymorphisms, RS1 and RS3, on relative promoter activity.

Overall, we observed a single association with *AVPR1A *in the Irish autism trio collection (rs11174815, corrected *P *= 0.01). Due to the very low minor allele frequency (minor allele frequency (MAF) = 0.015) and lack of observations for transmissions of the A allele, it is difficult to conclude the relationship between this marker and autism in the Irish autism sample from our study. Furthermore, a weak association signal was seen between the dichotomised RS1 polymorphism and individuals with autism in the Irish sample. The short alleles were overtransmitted in individuals with autism.

We performed an experimental examination of the effect of allele length of the RS1 and RS3 polymorphism on relative promoter activity in the luciferase gene reporter assay. For the RS1 variant we observed the shorter allele to show lower relative promoter activity. Previous studies have reported shorter RS1 polymorphisms show more activity in the left amygdala in a non-clinical sample [36]. Excessive amygdala activation during social interaction has been reported to be associated with increase anxiety and eventually social withdrawal [37]. These aligning results suggest shorter alleles of RS1 result in lower relative promoter activity and possibly reduced transcription of *AVPR1A*, increasing amygdala activity leading to social withdrawal.

Shorter alleles of the RS3 polymorphism also lead to lower relative promoter activity in the luciferase gene reporter assay. While the RS3 polymorphism was not shown to be associated with autism in the Irish autism sample, it has been shown previously to be associated with individuals with autism [17,18,38]. Knafo *et al. *[20] reported individuals with shorter variants of the RS3 polymorphism have lower hippocampal *AVPR1A *mRNA and shorter alleles lead to less prosocial behaviours than longer alleles. Potentially, shorter alleles of the RS3 polymorphism, which show lower relative promoter activity, lead to reduced levels of *AVPR1A *mRNA and lower levels of AVPR1A in cells. Decreased levels of AVPR1A potentially could result in less altruistic behaviours (Table 3).

**Table 3:** Results from previous studies on the arginine vasopressin receptor 1A gene (*AVPR1A*) RS3 polymorphism demonstrating the functionality of this region

*AVPR1A *RS3 repeat length classification	**Postmortem hippocampal mRNA levels **[20]	**Prepulse inhibition **[45]	**Dictator game and real money allocations **[20]	**Amygdala activation to face recognition **[21]	Relative transcriptional activity (current study)
Short	Down	Down	Down	Down	Down

Long	Up	Up (males)	Up	Up	Up

Up/down indicate the direction of effect. Adapted from Levin *et al. *[45].

In a recent study, the human RS1 polymorphism showed no differences in COS7 cells [39]. Furthermore, Hong *et al. *[39] repeated the experiment in the PC12 cell line with no difference shown; however expression levels were extremely low indicating a potential weak promoter in AVPR1A. The difference in results between this study and ours is not surprising given recent evidence that *cis*-acting variation differs between tissue and cell types [40-44]. Our study examines the enhancer effects of RS1 and RS3 in an appropriate and relevant cell type for consideration in the aetiology of autism, a neurological disorder. In the study undertaken here, RS1 and RS3 are examined as enhancers modulating a viral promoter SV40 instead of its own natural promoter. Gene reporter systems do not take into account the genomic or chromatin context of the elements tested, however it is encouraging that our data agree with postmortem mRNA levels as shown in Knafo *et al. *[20], although additional studies are warranted. While the genomic environment tested was an artificial construction without the use of the endogenous promoter, these data do indicate that the repeat elements influence the relative activity of a core promoter in neuronal cells.

## Conclusions

The results from both RS3 and RS1 luciferase gene reporter assays are intriguing, with potentially shorter variants of both RS3 and RS1 leading to decreases in *AVPR1A *mRNA. Our results align with the study undertaken on the *Microtus *microsatellites, which showed decreased microsatellite length contributes to decreased sociability [13]. This suggests a potential conservation of function across species highlighting the importance of these polymorphisms. However, the functionality of both polymorphisms in our study complicates any conclusions. A limitation of the gene reporter approach is the removal of additional regulatory mechanisms, which may affect the DNA region of interest, and in these particular polymorphisms, how these polymorphisms may interact with each other in the regulation of *AVPR1A *transcription. Our haplotype analysis between the dichotomised alleles of RS1 and RS3 showed no association in the Irish sample. Rigorous meta-analysis is currently not possible due to the use of different genotyping methodologies [45]. We have used the same genotyping primers as the Knafo *et al. *[20], Meyer-Lindenberg *et al. *[21] and Levin *et al. *[45] allowing us to draw conclusions easily across these studies. Future work examining the regulatory relationship between these two polymorphisms will be essential for better understanding of how they influence *in vivo *transcription of *AVPR1A *and ultimately how they potentially play a role in the aetiology of autism.

## Competing interests

The authors declare that they have no competing interests.

## Authors' contributions

KET and RJLA wrote the manuscript. KET performed the laboratory work and data analysis undertaken in this manuscript. KET, MJH and RJLA conceived the experiment. MJH and RJLA supervised design, laboratory methods, and data analysis. MJH, LEC, MG and LG aided in manuscript preparation. MG, LG and RJLA were involved in the supervision and conception of the experiment.

## References

[B1] Goodson JL Bass AH Social behavior functions and related anatomical characteristics of vasotocin/vasopressin systems in vertebrates Brain Res Brain Res Rev 2001 35 246 265 10.1016/S0165-0173(01)00043-1 11423156

[B2] Bales KL Plotsky PM Young LJ Lim MM Grotte N Ferrer E Carter CS Neonatal oxytocin manipulations have long-lasting, sexually dimorphic effects on vasopressin receptors Neuroscience 2007 144 38 45 10.1016/j.neuroscience.2006.09.009 17055176 PMC1774559

[B3] Ferris WF Naran NH Crowther NJ Rheeder P van der Merwe L Chetty N The relationship between insulin sensitivity and serum adiponectin levels in three population groups Horm Metab Res 2005 37 695 701 10.1055/s-2005-870580 16308839

[B4] Chakrabarti S Fombonne E Pervasive developmental disorders in preschool children: confirmation of high prevalence Am J Psychiatry 2005 162 1133 1141 10.1176/appi.ajp.162.6.1133 15930062

[B5] Boso M Emanuele E Politi P Pace A Arra M Ucelli di Nemi S Barale F Reduced plasma apelin levels in patients with autistic spectrum disorder Arch Med Res 2007 38 70 74 10.1016/j.arcmed.2006.08.003 17174726

[B6] Bielsky IF Hu SB Young LJ Sexual dimorphism in the vasopressin system: lack of an altered behavioral phenotype in female V1a receptor knockout mice Behav Brain Res 2005 164 132 136 10.1016/j.bbr.2005.06.005 16046007

[B7] Bielsky IF Hu SB Szegda KL Westphal H Young LJ Profound impairment in social recognition and reduction in anxiety-like behavior in vasopressin V1a receptor knockout mice Neuropsychopharmacology 2004 29 483 493 10.1038/sj.npp.1300360 14647484

[B8] Lim MM Bielsky IF Young LJ Neuropeptides and the social brain: potential rodent models of autism Int J Dev Neurosci 2005 23 235 243 10.1016/j.ijdevneu.2004.05.006 15749248

[B9] Young LJ Toloczko D Insel TR Localization of vasopressin (V1a) receptor binding and mRNA in the rhesus monkey brain J Neuroendocrinol 1999 11 291 297 10.1046/j.1365-2826.1999.00332.x 10223283

[B10] Insel TR Wang ZX Ferris CF Patterns of brain vasopressin receptor distribution associated with social organization in microtine rodents J Neurosci 1994 14 5381 5392 8083743 10.1523/JNEUROSCI.14-09-05381.1994 PMC6577077

[B11] Young LJ Winslow JT Nilsen R Insel TR Species differences in V1a receptor gene expression in monogamous and nonmonogamous voles: behavioral consequences Behav Neurosci 1997 111 599 605 10.1037/0735-7044.111.3.599 9189274

[B12] Hammock EA Young LJ Variation in the vasopressin V1a receptor promoter and expression: implications for inter- and intraspecific variation in social behaviour Eur J Neurosci 2002 16 399 402 10.1046/j.1460-9568.2002.02083.x 12193181

[B13] Hammock EA Lim MM Nair HP Young LJ Association of vasopressin 1a receptor levels with a regulatory microsatellite and behavior Genes Brain Behav 2005 4 289 301 10.1111/j.1601-183X.2005.00119.x 16011575

[B14] Hammock EA Young LJ Microsatellite instability generates diversity in brain and sociobehavioral traits Science 2005 308 1630 1634 10.1126/science.1111427 15947188

[B15] Thibonnier M Graves MK Wagner MS Chatelain N Soubrier F Corvol P Willard HF Jeunemaitre X Study of V(1)-vascular vasopressin receptor gene microsatellite polymorphisms in human essential hypertension J Mol Cell Cardiol 2000 32 557 564 10.1006/jmcc.2000.1108 10756113

[B16] Kim SJ Young LJ Gonen D Veenstra-VanderWeele J Courchesne R Courchesne E Lord C Leventhal BL Cook EH Jr Insel TR Transmission disequilibrium testing of arginine vasopressin receptor 1A (AVPR1A) polymorphisms in autism Mol Psychiatry 2002 7 503 507 10.1038/sj.mp.4001125 12082568

[B17] Wassink TH Piven J Vieland VJ Pietila J Goedken RJ Folstein SE Sheffield VC Examination of AVPR1a as an autism susceptibility gene Mol Psychiatry 2004 9 968 972 10.1038/sj.mp.4001503 15098001

[B18] Yirmiya N Rosenberg C Levi S Salomon S Shulman C Nemanov L Dina C Ebstein RP Association between the arginine vasopressin 1a receptor (AVPR1a) gene and autism in a family-based study: mediation by socialization skills Mol Psychiatry 2006 11 488 494 10.1038/sj.mp.4001812 16520824

[B19] Yang SY Cho SC Yoo HJ Cho IH Park M Yoe J Kim SA Family-based association study of microsatellites in the 5' flanking region of AVPR1A with autism spectrum disorder in the Korean population Psychiatry Res 2010 178 199 201 10.1016/j.psychres.2009.11.007 20452058

[B20] Knafo A Israel S Darvasi A Bachner-Melman R Uzefovsky F Cohen L Feldman E Lerer E Laiba E Raz Y Nemanov L Gritsenko I Dina C Agam G Dean B Bornstein G Ebstein RP Individual differences in allocation of funds in the dictator game associated with length of the arginine vasopressin 1a receptor RS3 promoter region and correlation between RS3 length and hippocampal mRNA Genes Brain Behav 2008 7 266 275 10.1111/j.1601-183X.2007.00341.x 17696996

[B21] Meyer-Lindenberg A Kolachana B Gold B Olsh A Nicodemus KK Mattay V Dean M Weinberger DR Genetic variants in AVPR1A linked to autism predict amygdala activation and personality traits in healthy humans Mol Psychiatry 2009 14 968 975 10.1038/mp.2008.54 18490926 PMC2754603

[B22] Pinto D Pagnamenta AT Klei L Anney R Merico D Regan R Conroy J Magalhaes TR Correia C Abrahams BS Almeida J Bacchelli E Bader GD Bailey AJ Baird G Battaglia A Berney T Bolshakova N Bölte S Bolton PF Bourgeron T Brennan S Brian J Bryson SE Carson AR Casallo G Casey J Chung BH Cochrane L Corsello C Functional impact of global rare copy number variation in autism spectrum disorders Nature 466 368 372 10.1038/nature09146 20531469 PMC3021798

[B23] Anney R Klei L Pinto D Regan R Conroy J Magalhaes TR Correia C Abrahams BS Sykes N Pagnamenta AT Almeida J Bacchelli E Bailey AJ Baird G Battaglia A Berney T Bolshakova N Bölte S Bolton PF Bourgeron T Brennan S Brian J Carson AR Casallo G Casey J Chu SH Cochrane L Corsello C Crawford EL Crossett A A genome-wide scan for common alleles affecting risk for autism Hum Mol Genet 19 4072 4082 10.1093/hmg/ddq307 20663923 PMC2947401

[B24] Purcell SM Wray NR Stone JL Visscher PM O'Donovan MC Sullivan PF Sklar P Common polygenic variation contributes to risk of schizophrenia and bipolar disorder Nature 2009 460 748 752 19571811 10.1038/nature08185 PMC3912837

[B25] Lord C Rutter M Le Couteur A Autism Diagnostic Interview-Revised: a revised version of a diagnostic interview for caregivers of individuals with possible pervasive developmental disorders J Autism Dev Disord 1994 24 659 685 10.1007/BF02172145 7814313

[B26] Lord C Risi S Lambrecht L Cook EH Jr Leventhal BL DiLavore PC Pickles A Rutter M The autism diagnostic observation schedule-generic: a standard measure of social and communication deficits associated with the spectrum of autism J Autism Dev Disord 2000 30 205 223 10.1023/A:1005592401947 11055457

[B27] Sparrow SS Cicchetti DV Diagnostic uses of the Vineland Adaptive Behavior Scales J Pediatr Psychol 1985 10 215 225 10.1093/jpepsy/10.2.215 4020603

[B28] Barrett JC Fry B Maller J Daly MJ Haploview: analysis and visualization of LD and haplotype maps Bioinformatics 2005 21 263 265 10.1093/bioinformatics/bth457 15297300

[B29] Dudbridge F Likelihood-based association analysis for nuclear families and unrelated subjects with missing genotype data Hum Hered 2008 66 87 98 10.1159/000119108 18382088 PMC2386559

[B30] Galimberti D Scarpini E Venturelli E Strobel A Herterich S Fenoglio C Guidi I Scalabrini D Cortini F Bresolin N Lesch KP Reif A Association of a NOS1 promoter repeat with Alzheimer's disease Neurobiol Aging 2008 29 1359 1365 10.1016/j.neurobiolaging.2007.03.003 17418914

[B31] Reif A Herterich S Strobel A Ehlis AC Saur D Jacob CP Wienker T Topner T Fritzen S Walter U Schmitt A Fallgatter AJ Lesch KP A neuronal nitric oxide synthase (NOS-I) haplotype associated with schizophrenia modifies prefrontal cortex function Mol Psychiatry 2006 11 286 300 10.1038/sj.mp.4001779 16389274

[B32] Reif A Jacob CP Rujescu D Herterich S Lang S Gutknecht L Baehne CG Strobel A Freitag CM Giegling I Romanos M Hartmann A Rösler M Renner TJ Fallgatter AJ Retz W Ehlis AC Lesch KP Influence of functional variant of neuronal nitric oxide synthase on impulsive behaviors in humans Arch Gen Psychiatry 2009 66 41 50 10.1001/archgenpsychiatry.2008.510 19124687

[B33] Reif A Kiive E Kurrikoff T Paaver M Herterich S Konstabel K Tulviste T Lesch KP Harro J A functional NOS1 promoter polymorphism interacts with adverse environment on functional and dysfunctional impulsivity Psychopharmacology (Berl) 2010 10.1007/s00213-010-1915-7 20589495

[B34] Purcell S Neale B Todd-Brown K Thomas L Ferreira MA Bender D Maller J Sklar P de Bakker PI Daly MJ Sham PC PLINK: a tool set for whole-genome association and population-based linkage analyses Am J Hum Genet 2007 81 559 575 10.1086/519795 17701901 PMC1950838

[B35] Gabriel SB Schaffner SF Nguyen H Moore JM Roy J Blumenstiel B Higgins J DeFelice M Lochner A Faggart M Liu-Cordero SN Rotimi C Adeyemo A Cooper R Ward R Lander ES Daly MJ Altshuler D The structure of haplotype blocks in the human genome Science 2002 296 2225 2229 10.1126/science.1069424 12029063

[B36] Meyer-Lindenberg A Kolachana B Gold B Olsh A Nicodemus KK Mattay V Dean M Weinberger DR Genetic variants in AVPR1A linked to autism predict amygdala activation and personality traits in healthy humans Mol Psychiatry 2009 14 968 75 10.1038/mp.2008.54 18490926 PMC2754603

[B37] Stein MB Goldin PR Sareen J Zorrilla LT Brown GG Increased amygdala activation to angry and contemptuous faces in generalized social phobia Arch Gen Psychiatry 2002 59 1027 1034 10.1001/archpsyc.59.11.1027 12418936

[B38] Israel S Lerer E Shalev I Uzefovsky F Reibold M Bachner-Melman R Granot R Bornstein G Knafo A Yirmiya N Ebstein RP Molecular genetic studies of the arginine vasopressin 1a receptor (AVPR1a) and the oxytocin receptor (OXTR) in human behaviour: from autism to altruism with some notes in between Prog Brain Res 2008 170 435 449 full_text 18655900 10.1016/S0079-6123(08)00434-2

[B39] Hong KW Matsukawa R Hirata Y Hayasaka I Murayama Y Ito S Inoue-Murayama M Allele distribution and effect on reporter gene expression of vasopressin receptor gene (AVPR1a)-linked VNTR in primates J Neural Transm 2009 116 535 538 10.1007/s00702-009-0219-8 19350216

[B40] Cowles CR Hirschhorn JN Altshuler D Lander ES Detection of regulatory variation in mouse genes Nat Genet 2002 32 432 437 10.1038/ng992 12410233

[B41] Koch O Kwiatkowski DP Udalova IA Context-specific functional effects of IFNGR1 promoter polymorphism Hum Mol Genet 2006 15 1475 1481 10.1093/hmg/ddl071 16600993

[B42] Sun C Southard C Witonsky DB Olopade OI Di Rienzo A Allelic imbalance (AI) identifies novel tissue-specific cis-regulatory variation for human UGT2B15 Hum Mutat 31 99 107 10.1002/humu.21145 19847790 PMC2922057

[B43] Wilkins JM Southam L Price AJ Mustafa Z Carr A Loughlin J Extreme context specificity in differential allelic expression Hum Mol Genet 2007 16 537 546 10.1093/hmg/ddl488 17220169

[B44] Zhang K Li JB Gao Y Egli D Xie B Deng J Li Z Lee JH Aach J Leproust EM Eggan K Church GM Digital RNA allelotyping reveals tissue-specific and allele-specific gene expression in human Nat Methods 2009 6 613 618 10.1038/nmeth.1357 19620972 PMC2742772

[B45] Levin R Heresco-Levy U Bachner-Melman R Israel S Shalev I Ebstein RP Association between arginine vasopressin 1a receptor (AVPR1a) promoter region polymorphisms and prepulse inhibition Psychoneuroendocrinology 2009 34 901 908 10.1016/j.psyneuen.2008.12.014 19195791

